# A bioinformatics pipeline for a tick pathogen surveillance multiplex amplicon sequencing assay

**DOI:** 10.1016/j.ttbdis.2023.102207

**Published:** 2023-05-27

**Authors:** Lynn M. Osikowicz, Andrias Hojgaard, Sarah Maes, Rebecca J. Eisen, Mark D. Stenglein

**Affiliations:** a Division of Vector-Borne Diseases, National Center for Emerging and Zoonotic Infectious Diseases, Centers for Disease Control and Prevention, Fort Collins, CO, United States; b Center for Vector-Borne Infectious Disease, Department of Microbiology, Immunology, and Pathology, College of Veterinary Medicine and Biomedical Sciences, Colorado State University, Fort Collins, CO, United States

**Keywords:** Tick-borne diseases, Tick surveillance, Next generation sequencing, Bioinformatics

## Abstract

The Centers for Disease Control and Prevention’s national tick and tick-borne pathogen surveillance program collects information to better understand the regional distribution, prevalence, and exposure risk of host-seeking medically important ticks in the United States. A recently developed next generation sequencing (NGS) targeted multiplex PCR amplicon sequencing (MPAS) assay has enhanced the detection capabilities for *Ixodes*-associated human pathogens found in *Ixodes scapularis* and *Ixodes pacificus* ticks compared to the routinely used real-time PCR assay. To operationalize the MPAS assay for the large number of tick surveillance submissions processed each year, a reproducible high throughput bioinformatics pipeline is needed. We describe the development and validation of the MPAS pipeline, a bioinformatics pipeline that identifies and summarizes amplicon sequences produced by the MPAS assay. This pipeline is portable and reproducible across different computing environments, and flexible by allowing modifications to input parameters, assay primer and reference sequences. The automation of the summary report, BLAST report, and phylogenetic analysis reduces the amount of time needed for downstream analysis. To validate this pipeline, we compared the analysis of a MPAS assay dataset consisting of 175 *I. scapularis* nymphs with the MPAS pipeline and previously published results analyzed with a CLC Genomic Workbench workflow. The MPAS pipeline identified the same number of positive ticks for *Anaplasma phagocytophilum* and *Babesia* species as the original analysis, but the MPAS pipeline provided enhanced sequencing resolution of *Borrelia burgdorferi* sensu lato co-infected samples. The reproducibility, flexibility, analysis automation, and improved sequence resolution of the MPAS pipeline make it well suited for a high throughput tick pathogen surveillance program.

## 1. Introduction

Tick-borne diseases account for the majority of all reported vector-borne disease cases in the United States (Rosenberg et al., 2018). The incidence of tick-borne diseases continues to increase, cases are reported over an expanding region, and novel tickborne disease agents continue to be identified (Eisen et al., 2017). To track changes in environmental risk factors for tick-borne diseases, the Centers for Disease Control and Prevention (CDC) established a national tick and tick-borne pathogen surveillance program in 2018. The program collects data to better understand the regional distribution, prevalence, and exposure risk of host-seeking medically important ticks (CDC, 2018; Eisen et al., 2017). The CDC provides pathogen testing support services to public health partners for *Ixodes scapularis* and *Ixodes pacificus* ticks. In the United States, these two tick species alone are responsible for transmitting up to seven known human pathogens including *Borrelia burgdorferi* sensu stricto (s.s.), the primary causative agent of Lyme disease, for which an estimated 476,000 Americans are treated each year (Eisen and Paddock, 2021; Kugeler et al., 2021). The other known human pathogens transmitted by *I. scapularis* include *Borrelia mayonii, Borrelia miyamotoi, Anaplasma phagocytophilum, Ehrlichia muris eauclarensis, Babesia microti*, and Powassan virus (Eisen and Paddock, 2021). In addition to *B. burgdorferi* s.s*., I. pacificus* can also transmit *B. miyamotoi and A. phagocytophilum* (Eisen and Paddock, 2021). There are eight other characterized *Borrelia* species found in *Ixodes* ticks in the U.S. that belong to the *B. burgdorferi* sensu lato (s.l.) complex (*B. americana, B. andersonii, B. bissettiae, B. californiensis, B. carolinensis, B. kurtenbachii, B. lanei*, and *B. maritima*), but their pathogenic potential is not well described ((Rudenko et al., 2011; Wolcott et al., 2021).

The existing tick surveillance testing algorithm consists of five multiplex TaqMan based real-time polymerase chain reaction (PCR) assays, which provide species level detection for the *Ixodes* spp. pathogens (Graham et al., 2018). Recent developments of a next generation sequencing (NGS) targeted multiplex PCR amplicon sequencing assay (MPAS) have shown comparable sensitivity and enhanced specificity to the TaqMan algorithm, while also requiring less input nucleic acid (Hojgaard et al., 2020). The MPAS assay amplifies sequences from four microbial genera that include pathogens transmitted by *Ixodes* species ticks: *Borrelia* spp., *Babesia* spp., *Ehrlichia* spp., and *Anaplasma* spp., and the tick actin gene, which acts as an internal control (Hojgaard et al., 2020). The final products are 150–350 bp amplicon sequences, which improves the detection capabilities to include a wider range of microbial species and detects co-infected ticks (Hojgaard et al., 2020). Compared with the existing TaqMan testing algorithm, the MPAS assay expands the wealth of microbial information that can be collected from tick-borne pathogen surveillance testing. However, to operationalize the assay to accommodate testing of more than 7000 tick submissions per year, a reproducible high throughput bioinformatics pipeline is needed to efficiently analyze the NGS data that is produced from the large number of ticks annually tested.

Here we describe the development and validation of the MPAS pipeline, a bioinformatics pipeline that identifies and summarizes amplicon sequences produced by the MPAS assay. A number of goals guided the design and implementation of the pipeline. The pipeline should be at least as accurate as existing methods. It should be portable and reproducible and be able to run on different computing environments, and results should not vary on different platforms. It should be able to efficiently analyze large numbers of datasets. It should be modifiable to add or remove targeted pathogens. It should output and log results for surveillance efforts and provide a starting point for future research on tick borne pathogens. This paper describes how the pipeline was developed to have these properties and how it was validated in comparison to existing testing protocols.

## 2. Materials and methods

### 2.1. MPAS pipeline development

The MPAS pipeline is implemented in the Nextflow workflow management language, version 20.10.0 (Di Tommaso et al., 2017). Nextflow pipelines offer a number of advantages including scripting flexibility and portability, as pipeline can be run on a local computer, cluster system, or cloud environment (Di Tommaso et al., 2017). To ensure portability to other computing environments, other software dependencies are handled using Singularity containers or Conda environments (Anaconda Software Distribution, 2020; Di Tommaso et al., 2017; Kurtzer et al., 2017). These environments and containers lock software versions to ensure reproducibility. The MPAS pipeline is available in the GitHub repository: CDCgov/tick_surveillance (github.com)

#### 2.1.1. MPAS pipeline user input

The pipeline is run from the command line using Nextflow. The user provides the MPAS pipeline with several input files (Table 1) that exist in default locations relative to the run directory, but those locations can be overridden as described in the pipeline documentation (CDCgov/tick_surveillance (github.com)).

The metadata file must contain the required columns listed in Table 1. An additional column titled ‘*Batch*’ can be included if it is desired to group samples together for analysis. Any other relevant metadata information can be included in the metadata file at the discretion of the user. These metadata will be output as part of the pipeline’s results.

The *primers.tsv, targets.tsv*, and *surveillance_columns.txt* files can be customized for the specific MPAS assay and include assay primer information, reference sequences, and the reported output information desired from the analysis (Table 1). The *targets* file contains the reference sequences and alignment parameters used to map sample reads to reference sequences. The user can indicate in the *reporting_columns* field if the read count or the species name should be used as the reported parameter in the surveillance report file. The user can indicate ‘NA’ in the *reporting_columns* field if the reference sequence should not be listed in the summarized surveillance report. The *surveillance_columns.txt* file indicates the information that will be included in the output surveillance report. This report displays a summarized view of the target microorganism calls for each MPAS pipeline run. To include a reference sequence in the summarized surveillance report, the following should be indicated on a single line in the *surveillance_columns.txt* file; the *reporting_column* entry from the *targets.tsv* file, tab spaced, and ‘Negative’, which indicates that this entry will be assessed for sufficient species read numbers. The entry will receive a ‘Positive’ or ‘Negative’ call in the sequencing report file if the species read number is greater than the minimum number of target reads.

#### 2.1.2. MPAS pipeline workflow

**Fig. 1.**
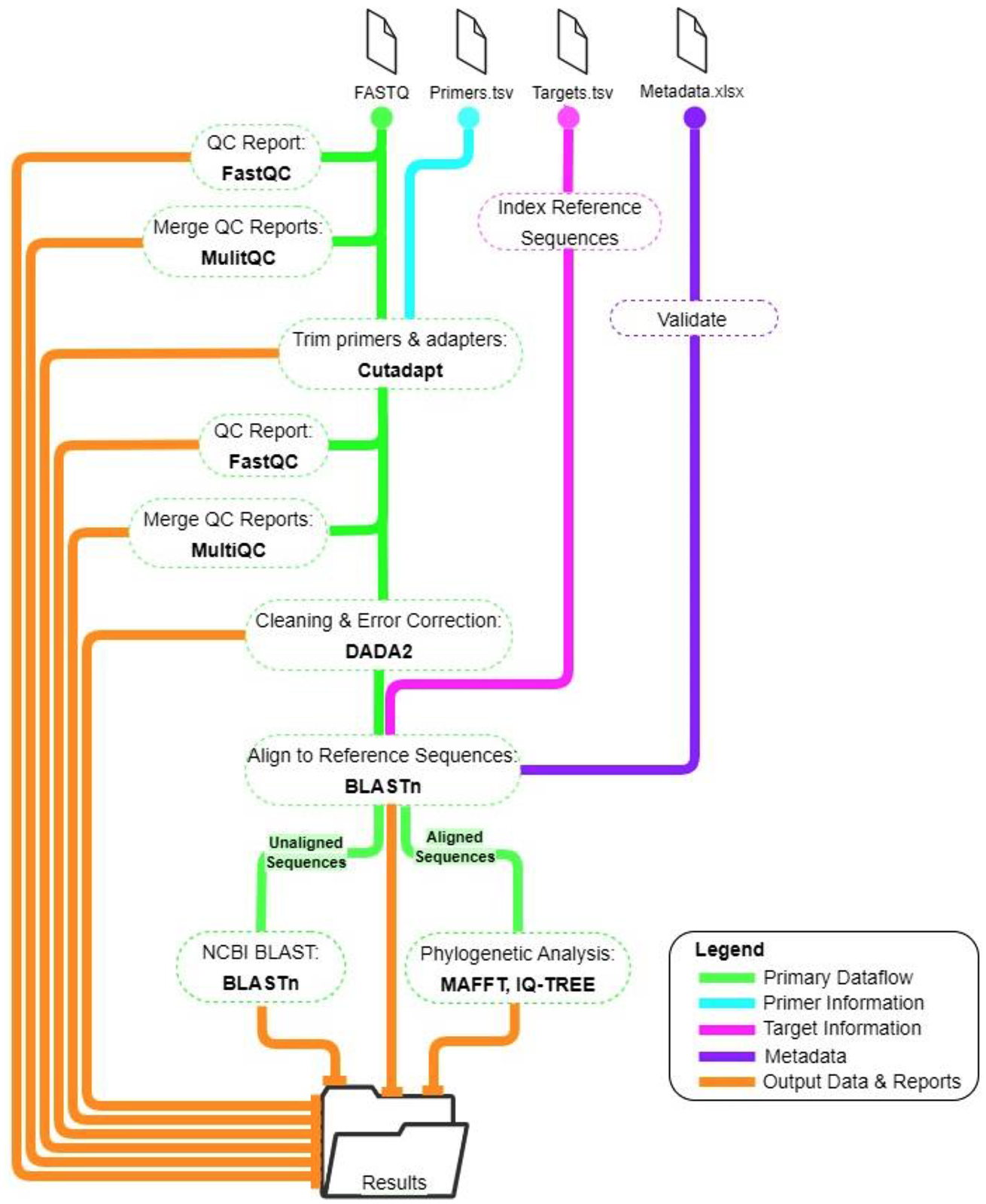
The MPAS Pipeline workflow. Solid lines indicate dataflow and dashed ovals indicate workflow processes. Analysis tools are in bold.

The sequencing analysis of the input FASTQ files occurs in nine primary workflow steps (Fig. 1) using open-source software tools (Table 2). First, the reference sequences from the *targets.csv* file are converted to individual FASTA files and then merged into one FASTA file. Next, the reference sequences are indexed which is used downstream for sequence alignment. The quality of input FASTQ files is then analyzed with FastQC version 0.11 and quality reports are merged with MultiQC version 1.1. Cutadapt version 3.5 is used to remove reads shorter than the set minimum read length (100 bp by default) and to trim primers specified in the *primers.tsv* file and Illumina Nextera adapters (Illumina, San Diego, CA, USA) from each read. Cutadapt will only keep reads that contain the correct primer pairs and primer orientation, which discards artifactual or off-target amplicons. The quality scores of the trimmed sequences are then reassessed and combined into one report.

Next, the reads are processed by DADA2 version 1.18. This tool performs error correction, merges paired reads, groups identical sequences (amplicon sequence variants, ASVs), and tabulates the number of read pairs for each ASV. The observed ASVs are aligned to reference sequences with configurable sequence similarity cutoffs using BLASTn version 2.10. The number of internal control (tick actin) reads and pathogen reads are analyzed for each sample. A sample is considered to have sufficient internal control reads if the log number of internal control reads for each sample is within three times the standard deviation of average log of internal control reads per batch. If batch groups are not set, then internal control read cut-off is calculated from all the samples in the analysis. The default minimum number of read pairs aligned to reference sequences required for a positive call is 50. Next, a phylogenetic analysis of the aligned sequences amplified from each assay primer is performed. The FASTA files created contain the appropriate reference sequence from the *targets.tsv* file and a representative observed sequence from identified sequences grouped by state, tick species, and life stage. A multiple sequence alignment is performed for each FASTA file using MAFFT version 7.508, a maximum likelihood tree is created with IQ-TREE version 2.2.0.3, and pdf files for each tree are generated with ToyTree version 2.0.5. The phylogenetic analysis is intended to provide a supplemental visual to the user to help quickly identify divergent sequences and is not used to classify species. The user should determine if this visual is pertinent to their specific assay, as not all genes have the same phylogenetic power.

Finally, any sequences that did not align to the input reference sequences are searched against the NCBI nucleotide database using BLASTn. This search can take advantage of a local installation of this database or can run remotely. This step is meant to characterize divergent sequences that are outside of the sequence similarity parameters thresholds set for each reference sequence and could be useful for identifying divergent or novel pathogens whose sequences are amplified by the assay primers.

#### 2.1.3. MPAS pipeline results

Pipeline output is placed by default in a results directory and consists of run reports, trimmed FASTQ files, FastQC and MultiQC reports, a sequencing report file, NCBI BLAST report, phylogenetic trees, and other intermediate output files from the DADA2 and reference sequence alignment analysis steps. The sequence report file in Excel format contains multiple tabs which include a summarized surveillance report, sequence information, metadata, and run parameter information. The *Testing Results* tab reports if samples had acceptable internal control reads and identifies if samples pass the minimum read cutoff values for any of the specified surveillance species. The *surveillance_counts* tab reports the total number read pairs observed for each species call in the *surveillance* tab. The *data_by_species* tab displays the reads identified as particular species based on the alignment and read abundance parameters for the MPAS pipeline run. The *all_data* tab contains all the unique sequences identified from each sample and the related alignment information. Finally, the *metadata* and *targets* tabs contain the input metadata and target information for the pipeline run.

### 2.2. Pipeline validation

#### 2.2.1. Procedure

To validate the MPAS pipeline, we analyzed the MPAS assay FASTQ files used in the original description of the MPAS assay by Hojgaard et al. (2020) and compared the MPAS pipeline results to the original analysis performed using the CLC Genomic Workbench (Qiagen, Germantown, MD, USA) (Hojgaard et al., 2020). These datasets are derived from 175 host seeking *I. scapularis* nymphs collected in Connecticut, USA. Briefly, the primary PCR reaction contained primers targeting the four genera associated with *Ixodes* transmitted human pathogens: *Borrelia* spp. (*flaB*, 335 bp), *Babesia* spp. (*18S*, 247 bp), *Anaplasma* spp. (*groEL*, 315 bp), *Ehrlichia* spp. (*groEL*, 315 bp), and tick actin (156 bp) (Hojgaard et al., 2020). After the primary PCR, each reaction was purified following the protocol described by Hojgaard et al. (2020) and indexed using the Nextera XT index kit (Illumina). Once the indexes were added, each reaction was purified, pooled, and sequenced following the manufacturer’s instructions using the MiSeq Reagent Kit v3 (600-cycle) (Illumina) on the MiSeq instrument (Illumina).

The CLC Workbench analysis was run with the customized workflow and reference sequences described by Hojgaard et al. (2020). The primary CLC workflow steps include, QC reports, merge overlapping read pairs, trim adapters, mapping observed reads to reference sequences, and de novo assembly of un-mapped reads (Hojgaard et al., 2020). The BLAST analysis of the de novo assembly and target microorganism calls were performed manually (Hojgaard et al., 2020). Samples were considered positive for a pathogen if there was a 10-fold increase in mapped reads above the negative controls (Hojgaard et al., 2020). The MPAS pipeline analyzed this dataset with the default minimum read length and read cut-off values described above. The same reference sequences used in the CLC analysis were also used in the MPAS pipeline analysis with the inclusion of additional tick actin reference sequences (Supplement A Table 1). The minimum percent identity and minimum percent aligned cutoffs for the internal tick control were set to 85% and 95%. The reference sequence cutoffs were set to 95% for minimum percent identity and 99% for minimum percent aligned. The maximum percent gaps was set to 5% for all reference sequences. NCBI BLAST was used to further categorize the reported ASV found in the samples. The MPAS pipeline analyzed this dataset twice to confirm that the pipeline was reproducible in different computing environments. The first analysis was initiated using the Conda environment setting on a local Linux workstation, and the second analysis was initiated using the Singularity setting on a Linux terminal in a high-performance computing environment. Supplement B contains the *primers.tsv, targets.tsv*, and *surveillance_columns.txt* files used for the MPAS pipeline analysis. All raw FASTQ files are available at NCBI BioProject ID PRJNA937278 (BioSamples: SAMN33395139-SAMN33395313).

## 3. Results

In total, 175 samples were analyzed with the MPAS pipeline and CLC Workbench. The MPAS pipeline results from each computing environments (Conda, Singularity) produced identical pathogen calls for each sample in the dataset. The median number of normalized reads per target for each computing environment can be seen in Supplement A Table 2. Since both environments produced identical results, the Singularity environment results will be used to further discuss the pipeline results described below.

After primer trimming, the MPAS pipeline reported an average per sequence Phred score above 28 for each R1 and R2 file. An average of 89% of reads (standard deviation = 13.4%) in the dataset were assigned to the reference sequences. We have observed that overloading of the Illumina run can produce reads with regions of low quality, particularly in the beginning of reads. The resulting N basecalls cause the MPAS pipeline to fail to recognize expected primer sequences, resulting in negative results. We suggest optimizing the loading concentration for any newly developed MPAS assay. The sequencing libraries for this study were optimized to 10 pM.

The MPAS pipeline analysis of the samples yielded similar results as the CLC analysis (Tables 3, 4). The notable exception was detection of multiple *B. burgdorferi* s.l. strain types of *B. andersonii* in three samples by the MPAS pipeline, while the CLC analysis did not separate *B. burgdorferi* s.l. strain types, and only identified a single *B. andersonii* sequence in each of the three *B. andersonii* positive samples. The MPAS pipeline reported four unique sequences in a single sample that were 99–100% similar to two *B. burgdorferi* s.l. strain types, *B. burgdorferi* SI-10 (GenBank Accession AF264883) (814 total reads) and *B. burgdorferi* BC-1 (GenBank Accession AF264898) (108 total reads), both of which are considered strain types of *B. andersonii* (Lin et al., 2004). A second sample contained four unique sequences that were 99–100% similar to *B. burgdorferi* SI-10 (1159 total reads), *B. burgdorferi* BC-1 (594 total reads), and *B. andersonii* 21,038 (786 total reads), and the third sample contained six unique sequences that were 99–100% similar to *B. burgdorferi* strain SI-10 (2529 total reads) and *B. burgdorferi* BC-1 (1362 total reads).

One sample did not pass the MPAS actin read cutoff value for acceptable DNA, which was not a requirement of the CLC workflow. The MPAS pipeline analysis identified a median of 13.0 (Range: 0–23) tick actin reads and a median of 0 (Range:0) target microorganism reads in the negative controls. The CLC analysis identified a median of 0 (Range: 0–6) targeted microorganism reads in the negative controls and the tick actin reads were not evaluated (Hojgaard et al., 2020).

## 4. Discussion

The new bioinformatics workflow improved sensitivity through enhanced detection of *B. burgdorferi* s.l. co-infections. The newer pipeline also offers enhanced efficiency through automation and improved reproducibility through use of workflow management software and software containers. The MPAS pipeline creates two primary output report files for a dataset: one containing a summarized report of target microorganism calls, and the other containing the BLAST report for unaligned sequences. These improvements in automation enable the MPAS assay to be used in a high-throughput surveillance program. By comparison, the CLC analysis contains automated primary workflow steps but requires manual target microorganism calls, BLAST search of the un-mapped reads, and the creation of summary reports. The MPAS pipeline eliminates the user time required for these manual steps which improves analysis efficiency and increases reproducibility of the analysis across datasets. An additional benefit of this pipeline is customization. The user can customize assay primer information, reference sequences, alignment parameters, and the summary report layout, making this a flexible workflow that can be adapted to other amplicon sequencing assays.

All the microorganisms that were identified in the CLC Workbench analysis were also identified in the MPAS pipeline analysis. However, the MPAS pipeline was able to further distinguish *B. burgdorferi* s.l. co-infections, which were not identified by the original CLC analysis. This result is likely attributable to the difference in the sequence assembly between the MPAS pipeline and CLC analysis. The CLC analysis collapses all sequences that are within the set minimum sequence similarity parameter to one contig, potentially inhibiting the taxonomic resolution depending on how broad the parameter is set. In contrast, the MPAS pipeline identifies ASVs, which can differ by as little as one base pair, and then aligns the observed ASVs to the reference sequences and reports the percent similarity to the reference sequence. This difference enables the MPAS pipeline to report all observed ASVs within the minimum percent similarity parameter, improving the taxonomic resolution. The primary objective of the CDC national tick surveillance program is to identify human pathogens transmitted by ticks. The ability to provide enhanced resolution on *B. burgdorferi* s.l. complex co-infections is crucial for tick-borne pathogen surveillance because not all *B. burgdorferi* s.l. species are known to cause disease in humans. Distinguishing pathogenic and non-pathogenic *B. burgdorferi* s.l. species provides a more accurate estimation of exposure risk and pathogen prevalence from tick surveillance data, thus improving public health messaging and intervention strategies.

The benefits of the MPAS pipeline along with the portability and reproducibility of Nextflow pipelines, makes this pipeline an ideal analysis tools for high throughput tick and tick-borne pathogen surveillance programs. Through improvements in specificity, the MPAS assay and its associated bioinformatics pipeline improve assessments of acarological risk and aid in tickborne pathogen discovery.

## Supplementary Material

primers

sur_col

supA

targets

## Figures and Tables

**Table 1:** The required input files for the MPAS pipeline.

Required input files	Format	Required information
metadata	tsv	Column names: Index, Pathogen_Testing_ID, CSID, State, Morphological_Ectoparasite_Genus_Species, Lifestage
primers	tsv	Forward primer name, forward primer sequence, reverse primer name, reverse primer sequence
targets	tsv	Reference sequence name, species, primer name, reporting columns, minimum percent identity, minimum percent aligned, max percent gaps, internal control designation, sequence
surveillance_columns	txt	Column name entry must match the reporting column from targets.tsv file
Sequence files	FASTQ	Demultiplexed paired-end FASTQ files from one or more paired-end Illumina 500–600 cycle sequencing runs

**Table 2:** Analysis software used in the MPAS pipeline.

Software	Version	Conda Channel	Refs.
**BLAST**	2.10.*	Bioconda	Altschul et al., 1990; Camacho et al., 2009
**Cutadapt**	3	Bioconda	Martin, 2011
**DADA2**	1.18.*	Bioconda	Callahan et al., 2016
**FastQC**	0.11.*	Bioconda	Andrews, 2010
**IQ-TREE**	2.2.0.3	Bioconda	Minh et al., 2020
**MAFFT**	7.508	Bioconda	Katoh and Standley, 2013
**MultiQC**	1.1	Bioconda	Ewels et al., 2016
**Nextflow**	20.10.*	Bioconda	Di Tommaso et al., 2017
**Numpy**	1.22.*	Anaconda	Harris et al., 2020
**Openpyxl**	3.0.10	Anaconda	Eric Gazoni and Charlie Clark, 2022
**Pandas**	1.4.4	Anaconda	McKinney, 2010
**R-BASE**	4.0.*	Conda-forge	R Core Team, 2021
**R-dt**	0.17	Conda-forge	Yihui Xie et al., 2022
**R-openxlsx**	4.2.*	Conda-forge	Schauberger and Walker, 2022
**R-readxl**	1.3.*	Conda-forge	Wickham et al., 2019b
**R-Tidyverse**	1.3.*	Conda-forge	Wickham et al., 2019a
**ToyTree**	2.0.5	Conda-forge	Eaton, 2020
**XS**	1.0.*	Conda-forge	Pratas et al., 2014

* Indicates the latest update within the version number.

**Table 3:** Comparison of the identified targets detected 175 *I. scapularis* nymphs analyzed with the CLC Genomic Workbench (Qiagen) and the MPAS Pipeline. The CLC analysis data was originally reported in Hojgaard et al. (2020).

Identified Microorganism	Number of positive I. scapularis* nymphs (%) of 175 tested	
	CLC Analysis	MPAS Pipeline Analysis
*A. phagocytophilum*	5 (2.9)	5 (2.9)
*Ba. microti*	16 (9.1)	16 (9.1)
*Ba. odocoilei*	21 (12.0)	21 (12.0)
*B. burgdorferi s.s*.	29 (16.6)	29 (16.6)
*B. miyamotoi*	4 (2.3)	4 (2.3)
*B. andersonii*	4 (2.3)	4 (2.3)
Acceptable DNA^a^	NA	174 (99.4)

a Acceptable tick actin reads were not evaluated in the CLC analysis.

* Tick identification was based on morphology.

**Table 4:** Comparison of the co-infections detected in the 175 *I. scapularis* nymphs analyzed with the CLC Genomic Workbench and the MPAS Pipeline. The CLC analysis data was originally reported in Hojgaard et al. (2020).

Identified Co-infections	Number of positive I. scapularis* nymphs (%) of 175 tested	
	CLC Analysis	MPAS Pipeline Analysis
*B. burgdorferi s.s*. + *B. miyamotoi*	1 (0.6)	1 (0.6)
*B. burgdorferi s.s*. + *Ba. microti*	10 (5.7)	10 (5.7)
*B. burgdorferi s.s*. + *Ba. odocoilei*	2 (1.1)	2 (1.1)
*B. burgdorferi s.s*. + *A. phagocytophilum*	1 (0.6)	1 (0.6)
*Ba. microti* + *A. phagocytophilum*	1 (0.6)	1 (0.6)
*B. burgdorferi s.s*. + *A. phagocytophilum* + *Ba. microti* + *Ba. odocoilei*	1 (0.6)	1 (0.6)

* Tick identification was based on morphology.

## Data Availability

The github repository link has been included in the manuscript.
