# Supplementary material for: A bioinformatics pipeline for a tick pathogen surveillance multiplex amplicon sequencing assay
Source: Ticks Tick Borne Dis. Author manuscript; Available in PMC 2024 Feb 20. (PMC10878300; doi:10.1016/j.ttbdis.2023.102207)
Supplement: supA [file NIHMS1967886-supplement-supA.docx]

Table 1: Reference sequences used in the MPAS pipeline and CLC Workbench analysis.

>*Ixodes scapularis* actin gene GenBank accession AF426178 GGCCGTGCTCTCCCTGTACGCCTCCGGTCGTACCACGGGTATCGTGCTCGACTCCGGCGATGGCGTCTCCCACACCGTCCCCATCTACGAAGGGTACGCCCTGCCCCACGCCATCCTCCGTCTGGACTTGGCCGGCCGGGACCTGACCGACTACCTG

>*Ixodes scapularis* actin-5C. GenBank accession XM_029977298

GGCCGTGCTGTCCCTGTACGCGTCCGGCCGTACTACGGGCATCGTGCTCGACTCCGGCGATGGCGTCTCCCACACCGTGCCCATCTACGAGGGGTACGCCCTGCCGCACGCCATCCTCAGGCTGGACCTCGCCGGCCGGGACCTCACAGACTATCTC

>*Anaplasma phagocytophilum* strain JM. GenBank accession CP006617: 237405-237719

AATGATAAGGTGGGAGATGGAACTACTACATGCTCCATACTAACGGCAAAAGTGATTGAAGAAGTCTCAAAAGCGAAAGCTGCTGGATCTGATATTGTTAGCATAAAGAATGGTATTCTCAAGGCTAAGGAAGCGGTTCTTACAGCGCTTATGTCTATGAGACGTGAAGTAGAAGAAGACGAAATTGCACAAGTTGCAACATTGTCTGCGAATGGAGACAAGAACATAGGAAGTAAGATTGCACAGTGTGTTAAAGAAGTCGGTAAAGACGGTGTTATAACTGTTGAAGAAAGCAAAGGCTTCAAGGATCTAGAA

>*Ehrlichia* sp. ELMA. GenBank accession KU214846:372-689

AATGATAAAGTTGGTGATGGAACAACTACATGTTCTATTTTAACTGCAAAAGTTATAGAAGAAGTATCTAAAGCTAAAGCTGCTGGAGCAGATATTGTATGTATTAAAGAAGGTGTATTAAAAGCTAAAGAAGCTGTGCTAGAAGCATTAATGTCTATGAAGCGTGAAGTATTGTCTGAAGAGGAGATTGCTCAAGTTGCTACTATTTCAGCTAATGGAGATAAAAACATAGGTAGTAAAATTGCTCAATGTGTTCAAGAAGTTGGTAAAGATGGAGTTATTACAGTTGAAGAAAGTAAGGGATTCAAAGAATTGGAT

>Candidatus *Ehrlichia khabarensis* strain m3. GenBank accession KR063139:410-727

AATGATAAAGTTGGAGATGGAACAACTACATGTTCTATTTTGACAGCAAAAGTAATAGAAGAAGTATCTAAAGCTAAAGCTGCTGGTGCAGATATTGTATGTATTAAAGAAGGTGTATTAAAAGCTAAAGAAGCTGTACTAGAAGCTTTAATGTCAATGAAACGTGAAGTATTGTCTGAAGAAGAAATAGCACAAGTTGCTACTATCTCTGCTAATGGAGATAAAAATATAGGTAGTAAGATAGCACAATGTGTTCAGGAAGTTGGTAAAGATGGTGTTATTACAGTAGAAGAAAGTAAAGGGTTTAAAGAACTGGAT

*>Babesia microti* isolate Giresun 2010-30. GenBank accession MH523097: 43-317

TATTAAAGTTGTTGCAGTTAAGAAGCTCGTAGTTGAATTTCTGCCTTGTCATTAATCTCGCTTCCGAGCGTTTTTTTATTGACTTGGCATCTTCTGGATTTGGTGCCTTCGGGTACTATTTTCCAGGATTTACTTTGAGAAAACTAGAGTGTTTCAAACAGGCATTCGCCTTGAATACTACAGCATGGAATAATGAAGTAGGACTTTGGTTCTATTTTGTTGGTTATTGAGCCAGAGTAATGGTTAATAGGAGCAGTTGGGGGCATTCGTATTTA

*>Babesia odocoilei* strain CN18-5A70A*.* GenBank accession MK620851: 44-290

TATTAAACTTGTTGCAGTTAAAAAGCTCGTAGTTGAATTTCTGCGTCACCGTATTTTGACTTTTGTCGACTGTCGGTTTCGCTTTTGGGATTTATCCCTTTTTACTTTGAGAAAATTAGAGTGTTTCAAGCAGACTTTTGTCTTGAATACTTCAGCATGGAATAATAGAGTAGGACTTTGGTTCTATTTTGTTGGTTTGTGAACCTTAGTAATGGTTAATAGGAACGGTTGGGGGCATTCGTATTTA

*>Borrelia andersonii* strain 21038. Culture from internal reference collection

AAAAATTAACACACCAGCATCACTTTCAGGATCTCAAGCTTCTTGGACCCTAAGAGTTCATGTTGGAGCAAACCAAGATGAAGCTATTGCTGTAAATATTTATGCAGCTAATGTAGCAAATCTTTTTTCTGGTGAGGGAGCTCAAACTGCTCAGGCTGCACCTGTTCAAGAGGGTATTCAACAGGAAGGAGCTCAACAACCAGCACCTGCTACAGCACCTTCTCAAGGCGGAGTTAATTCTCCTGTTAATGTTACAACTACAGTTGATGCTAATACATCACTTGCTAAAATAGAAAATGCTATTAGAATGGTAAGTGATCAAAGAGCGAATTTAG

>*Borrelia burgdorferi* strain B31_NRZ. GenBank accession CP019767: 147835-148211 AAAAATTAACACACCAGCATCACTTTCAGGGTCTCAAGCGTCTTGGACTTTAAGAGTTCATGTTGGAGCAAACCAAGATGAAGCTATTGCTGTAAATATTTATGCAGCTAATGTTGCAAATCTTTTCTCTGGTGAGGGAGCTCAAACTGCTCAGGCTGCACCGGTTCAAGAGGGTGTTCAACAGGAAGGAGCTCAACAGCCAGCACCTGCTACAGCACCTTCTCAAGGCGGAGTTAATTCTCCTGTTAATGTTACAACTACAGTTGATGCTAATACATCACTTGCTAAAATTGAAAATGCTATTAGAATGATAAGTGATCAAAGGGCAAATTTAG

>*Borrelia miyamotoi* strain RI13-2395. Culture from internal reference collection

AAAAATTAACACACCAGCATCATTAGCTGGAACACAAGCTTCATGGACATTGAGAGTACATGTGGGTGCAAATCAGGATGAAGCAATTGCTGTCAATATTTATGCAGCTAATGTTGCAAATCTTTTTAATGGAGAAGGTGCTCAAGCCGCTCCAGCTCAAGAGGGAGCACAACAGGAGGGAGTTCAAGCAGCTCCAGCTCCAGCAGCCGCTCCAGTTCAAGGTGGAGTTAATTCTCCAATTAATGTTACAACTGCTATTGATGCTAATATGTCACTTTCAAAGATCGAAGATGCTATTAGAATGGTAACTGATCAAAGAGCAAATCTTG

>*Borrelia mayonii* strain MN14-1420. GenBank accession CP015780:148946-149322 AAAAATTAACACACCATCATCACTTTCAGGGTCTCAAGCTTCTTGGACCTTAAGAGTTCATGTTGGAGCAAATCAAGATGAAGCTATTGCTGTAAATATTTATGCAGCTAATGTTGCAAATCTTTTTTCTGGTGAGGGAACTCAAACTGCTCAGGTTGCGCCTGTTCAAGAAGGTGCTCAACAGGAAGGAGCTCAACAGCCAGCACCTGCTACAGCACCTTCTCAAGGCGGAGTTAATTCTCCTGTTAATGTTACAACTACAGTTGATGCTAATACATCACTTGCTAAAATAGAAAATGCTATTAGAATGATAAGTGATCAAAGAGCAAATTTAG

Table 2: The median number of normalized reads per target genus for the samples scored as positive with the MPAS pipeline run in the Conda and Singularity computing environments.

| Organism | Target | Computing Environment | | | |
| --- | --- | --- | --- | --- | --- |
|  |  | Singularity | | Conda | |
|  |  | Median Normalized Reads | Range | Median Normalized Reads | Range |
| *Borrelia spp.* | *flaB* | 2,282 | 193-9,520 | 2,282 | 193-9,520 |
| *Babesia spp.* | *18S* | 4,634 | 580-7,988 | 4,634 | 580-7,988 |
| *Anaplasma spp.* | *groEL* | 3,923 | 2,107-6433 | 3,923 | 2,107-6433 |
| *Ixodes scapularis* | *Actin* | 5,755 | 194-10,676 | 5,755 | 194-10,676 |
